# Supplementary material for: Chromothripsis during telomere crisis is independent of NHEJ, and consistent with a replicative origin
Source: Genome Res. 2019 May;29(5):737–49. doi: 10.1101/gr.240705.118 (PMC6499312; doi:10.1101/gr.240705.118)
Supplement: Supplemental Material [file supp_gr.240705.118_Supplemental_file_1.zip › contigs/annotated_contigs/DB55/contig.2.DB55_length_690_mean_cov_5.59710144928.docx]

**DB55_length_690_mean_cov_5.59710144928**

```
CTTCACTCAGTTTGTGGTGAATGCTTCCAGGCCTTGGATTCACCGTTCATGGCAGTGGGCTCCCCTCTGGCCCAGGGCAGGTCCATAAA
 >chr18:31880914-31881239 - E=9e-184
TGTCATCTAAGAGCCAAGGCCTAAAATTAAAGACCCTAAGAGCTCATACGGTGCTCTATTCCACTATGGCTGAGCTCATACCTGAAGCC

AGCATGTCTCAAAATCTCACTGAAAGCCCATGGCTTACTATCTGCAAGTATTGCTGCTTGCTCTTCAGGGTACAAGAGCTCTTCAGTCA

GCAGGTGATGAATCTTGCCAGGATTGCACCCTTCCTTTAAGGCAGTAGGTTCCC|TTCT|TATTGCTCGTTAACATTCCTTTCCTTCCG
                                                       >chr18:31882581-31882950 - E=3e-
ATTGAATAACTCCCTTTAGCATTTCTTATAAGATGCAGCTAGTGTTGATGAAATCCATCAGCTCTTGTTGATGAAATCCATCAGCTCCC
207
AAACAAGGGAATTCAAACATTCCCTCGTGTTTGAAGGATATTTTCACTGGATATATTATTCTAGGGTAAATTTTTTTTTTCCTCCAGCA

CTTTAAGTTGTCATACCACTCTCTCATGGCCTTTAAGGTTTGCTCTGAAAAGTGTGCTGCCAGATATATTGGAGCTCCATTGTATGGCA

CTTGCTTCCTTTCTCTTGCTGCTTTTCCTTTCTTTATTCTTGACCTTTAAGAGTTTGGTTATTAAAAGC
```
